# Eco-Friendly Fire-Retardant Coating on Cotton Using Layer by Layer Deposition Technique

**DOI:** 10.3390/molecules29245976

**Published:** 2024-12-18

**Authors:** Hamid Hassan, Zeeshan Ur Rehman, Bon Heun Koo

**Affiliations:** 1 Department of Materials Science and Engineering, Changwon National University, Changwon 51140, Gyeongsangnam-do, Republic of Korea; hamidhassan774@gmail.com; 2 College of Mechatronic Engineering, Changwon National University, Changwon 51140, Gyeongsangnam-do, Republic of Korea

**Keywords:** thermal properties, layer by layer, clay, eco-friendly, UL-94, fire retardant

## Abstract

Fire hazards are an increasing concern in several high-tech industries of public importance, particularly where textile fabrics are used in abundance. In this study, a novel layer by layer deposition method was utilized to develop a fire-retardant coating on cotton fabric. The method involves a hybrid cationic solution consisting of chitosan and branched polyethyleneimine, while bentonite clay was used as the anionic species. The treated fabric was characterized using SEM, VFT, and attenuated total reflection Fourier transform infrared spectroscopy (FTIR). SEM and EDS profiling confirmed the successful deposition of the (BPEI/CH + BNT) species on the surface of the cotton fabrics. FTIR analysis shows changes in chemical composition between the uncoated and coated samples, as confirmed by modifications in peaks at 3621 cm^−1^, 1023.3 cm^−1^, 1631 cm^−1^, and 614.8 cm^−1^. Finally, the thermal degradation behavior of pre-coated and post-coated samples was evaluated using thermogravimetric (TGA) analysis within a temperature range of 25 °C~700 °C, where the highest residue of ~19.83% was observed at 700 °C for the D-BPCB-30BL sample, signifying highly improved thermal stability compared to uncoated cotton.

## 1. Introduction

Cotton is one of the most essential natural fabrics that meets the fundamental requirements for luxury and comfort in human livelihood. It has been utilized extensively across various industrial sectors, including the apparel, electronic, and aerospace industries due to its softness, exceptional air permeability, biocompatibility, and renewable properties [1,2]. However, exposure to flame can cause immediate fire spread, leading to severe economic and physical losses [3]. The low thermal stability of pure cotton fabric is due to its weak fire-retardant properties, such as a low limiting oxygen index (LOI) of ~18% and a relatively low ignition temperature (360–425 °C), which make it highly flammable [4]. Therefore, to address this issue of high flammability, several fire-retardant coatings and finishing treatments have been applied to the surface of cotton fabric [5]. For instance, the use of the sol–gel process [6], plasma-assisted coatings [7], and the layer by layer technique has shown significant improvement in fire-retardant properties [8,9]. Among these coating techniques, the layer by layer (LbL) deposition method has been found to be an efficient, relatively convenient, and a molecular-level approach for producing highly homogenous and commercially scalable flame retardant coatings on cotton fabrics [10]. The layer by layer deposition method is environmentally friendly, allowing the deposition of novel fire-retardant materials on cotton [11]. Over the last two decades, researchers have studied the layer by layer deposition technique to investigate its use as a dynamic approach to manufacturing multilayer thin films [12].

The LbL method is a water-based deposition technique that involves depositing opposite charges on a substrate by alternately dipping it into a positively and negatively charged system or solution. Bilayers (BL) are formed due to strong electrostatic attraction [13], covalent bonding [14], hydrogen bonding [15], acceptor/donor interactions [16], and many other host–guest pairs [17] between layers. These interactions facilitate the deposition of thin films on different substrates. Considering the above discussion, the development of fire-retardant fabrics is a crucial research area for materials chemists [18].

A comprehensive review of the literature on fire-retardant research reveals that the most effective fire-retardant coatings include halogen compounds such as chlorine and bromine. These compounds have been extensively employed in the fire-retardant treatment of cotton fabrics. While highly effective in enhancing flame resistance, these flame retardants pose significant risks to both human health and the environment [19,20]. Consequently, it is essential to replace these hazardous materials with eco-friendly and non-toxic alternatives. Researchers are encouraged to focus on developing new fire-retardant coatings with minimum adverse effects on human health and the environment. Among the promising alternatives are bio-based compounds, such as chitosan, which is derived from crustacean shells. It contains a polyhydroxy structure and releases nitrogen and carbon residue at higher temperatures, contributing to its fire-retardant properties [21,22,23,24]. Another potential alternative is bentonite clay, known for its superior thermal and mechanical properties, lightweight nature, environmental-friendliness, and natural abundance. Bentonite is a phyllosilicate mineral characterized by a structure, in which an octahedral alumina sheet is sandwiched between two tetrahedral silica sheets. This unique structure facilitates hydrogen bonding, enhancing its interfacial interactions with various organic and inorganic matrices [25,26]. Several studies have reported the effective role of bentonite as a fire retardant [27]. Additionally, the use of bentonite clay in synergy with other bio-based compounds such as chitosan could further enhance the fire-retardant properties of the coatings while maintaining an eco-friendly profile.

In the current study, the fire-retardant composite D-BPCB was synthesized using a sequential LbL assembly method. It was anticipated that the use of a novel positively charged hybrid solution of BPEI/CH and a negatively charged bentonite clay solution could promote the formation of thermally stable carbonaceous structures and minimize the emission of flammable volatiles gases. Characterization further confirms the enhancement of effective flame suppression, indicating its potential effectiveness in improving fire safety.

## 2. Result and Discussion

### 2.1. Surface Morphology

**Figure 1:**
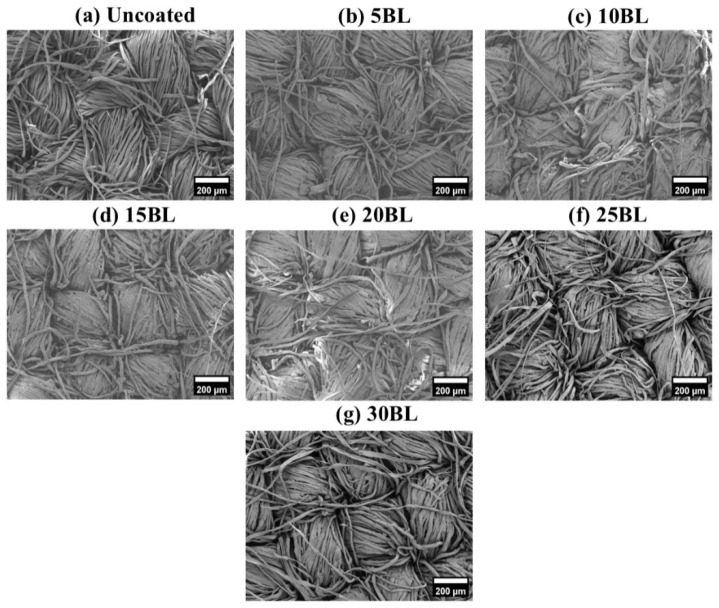
LV-SEM images of the uncoated and LbL-coated samples.

The surface morphology of the coated and uncoated samples can be seen in Figure 1a–g, whereas the detailed surface structure of each sample can be seen in the inset images.

**Figure 2:**
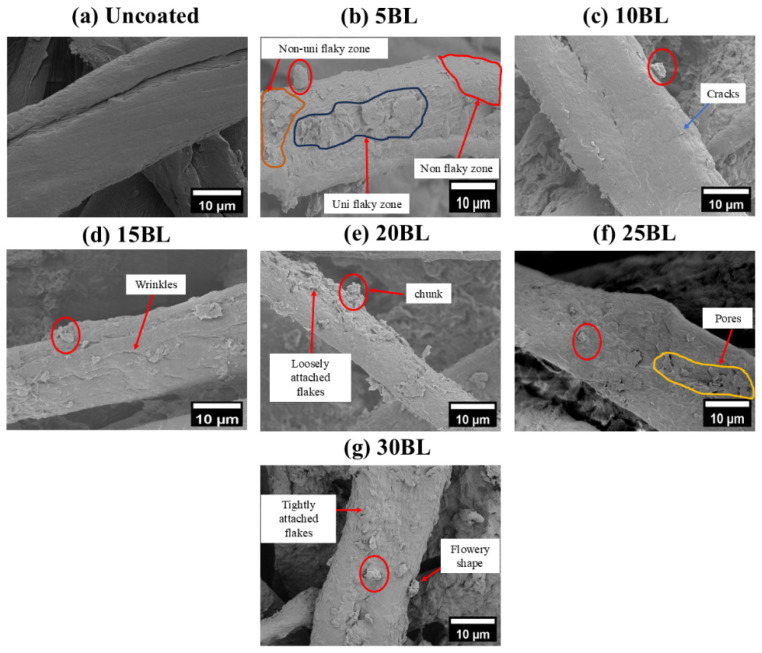
LV-SEM images of the uncoated and LbL-coated samples.

**Figure 3:**
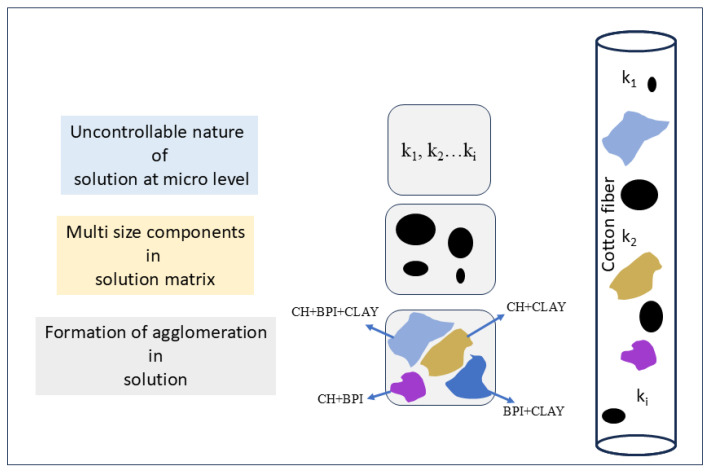
Schematic diagram of non-uniform coating.

**Figure 4:**
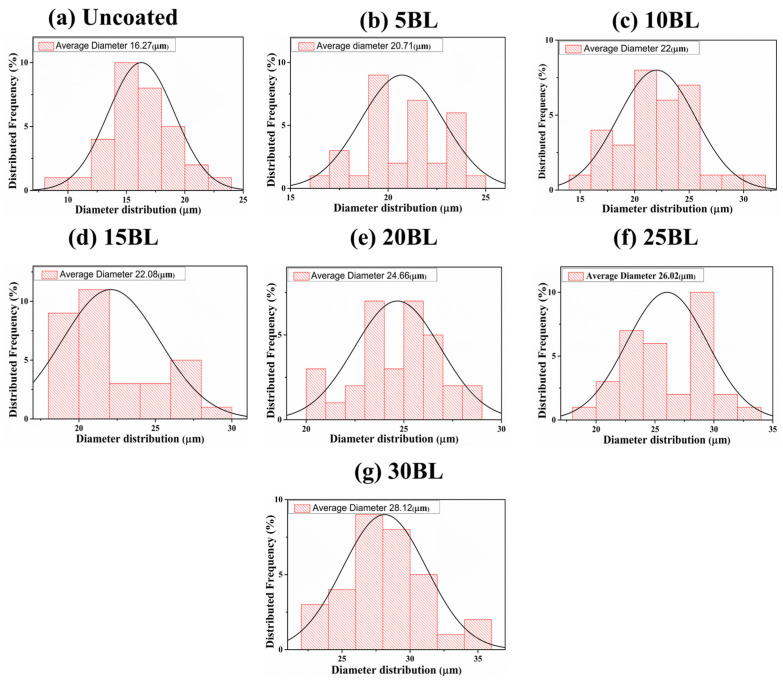
Diameter thickness distribution of coated samples.

The SEM images revealed a significant morphological difference between untreated and coated cotton fabric samples, as shown in Figure 1a–g. It can be clearly seen that the fibers of the untreated cotton fabric exhibit a compact and smooth surface morphology [28]. However, after the coating process, the fiber morphology changed, becoming coarser. Furthermore, a collectively uniform coating was observed on all the coated samples, although a non-uniform layered structure can be observed on individual fibers of the coated samples, as can be seen in Figure 2a–g. This may have resulted from three possible reasons. The first is the uncontrollable nature of chemical reactions at the microlevel, with different rates of chemical reaction (k_1_, k_2_, …, k_i_) on each individual site of the fiber. The second is the presence of multi-sized components within the solution matrix, where the size of nanoparticles significantly influences their adhesion to fibers. Larger nanoparticles tend to form agglomerates that can be easily washed away, while smaller particles penetrate deeper into the fabric and adhere more strongly [29]. The third is the formation of agglomeration within the solution matrix, leading to layers of varying thickness, as illustrated in the schematic diagram in Figure 3. However, this layered structure is not observed in any individual fiber of the untreated samples, confirming the successful deposition of the coating. Additionally, an increase in the diameter of cotton fibers is noted as the number of deposited layers increases, as shown in Figure 4a–g.

As the number of processing layers increases, a notable rise in surface roughness can be observed. In addition, the surface morphology can be classified into three zones: (a) non flakey zones, (b) non-uniform flakey zones that are caused by clay flakes partially submerged by the cationic layer, and (c) uniformly attached flakey zone where clay flakes are fully submerged by the cationic layer. The presence of wrinkles between the individual cotton fibers suggests the aggregation of clay particles due to interaction with electrolytes. Additionally, various features, such as chunks, loosely attached flakes, tightly attached flakes, and cracks in the coated layers can be seen on the individual fibers, as shown in Figure 2a–g. These features could have been caused as a result of agglomeration [30]. Loosely attached agglomerates with a flowery structure, formed from the combination of particles and clay flakes, can also be observed in Figure 2. This suggests that polyelectrolytes facilitate multi-cross linking through electrostatic interactions among the CH-BPEI/BNT and cotton fabrics, particularly when a high number of bilayers is assembled. Despite the modification, the LbL-assembled cotton maintained a similar hand feel with its corresponding original cotton fabric.

**Figure 5:**
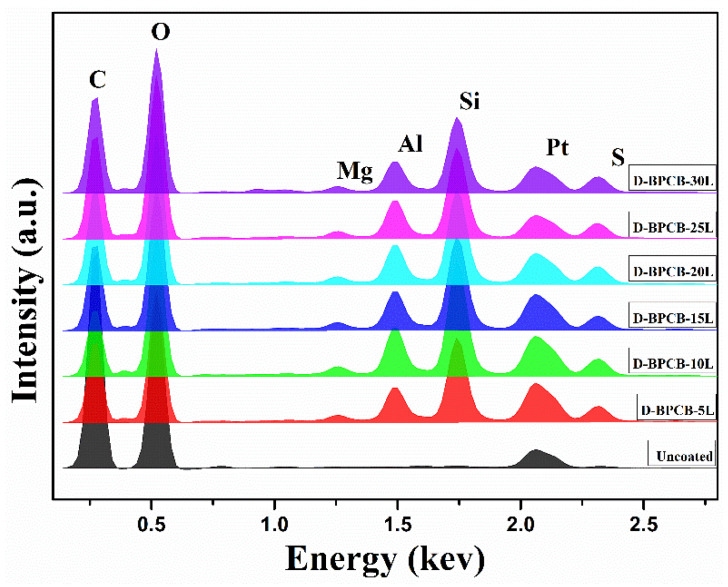
EDX analysis of the coated specimens.

To determine the elemental composition of the coating layers, energy-dispersive X-ray spectroscopy (EDX) was acquired for all coated samples, as illustrated in Figure 5. The EDX spectra revealed that the coated samples, each with different numbers of deposited layers, exhibited different percentages of trace elements, including Na, Mg, Al, and Si. These elements originated from the clay electrolyte solution. The analysis highlighted that the concentration of these elements plays a pivotal role in influencing the thermal stability and flame resistance of the coated fabrics. The presence of these clay-derived elements in the EDX profile suggests that the fire-retardant performance of the coated cotton fibers is significantly enhanced compared to the uncoated samples. The enhanced fire-retardant properties can be contributed to the sum effect of these elements, which form a protective barrier on the coated fabric surface. During combustion, an elemental coating of SiO_2_ is formed, which acts as an insulating layer to further slow down the degradation process by trapping the combustible gases [31].

### 2.2. FTIR Analysis

**Figure 6:**
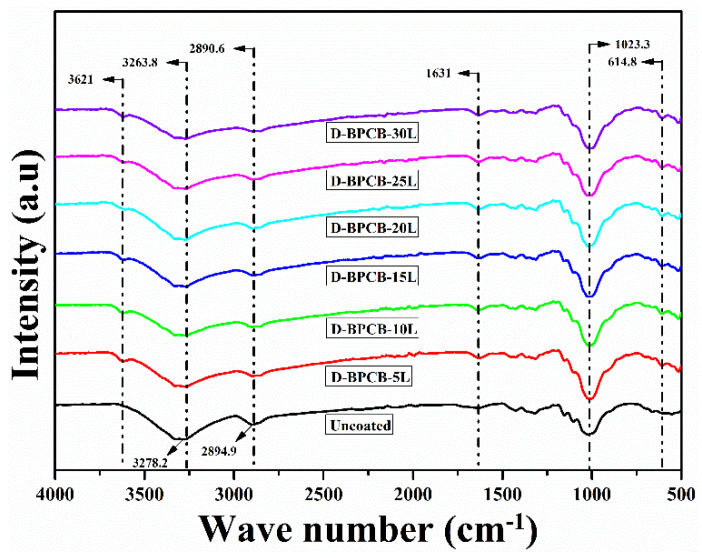
ATR-FTIR profile of the uncoated and coated cotton samples.

The FTIR spectra of both untreated and treated cotton samples were obtained, as shown in Figure 6. These were recorded in the spectral range of 500–4000 cm^−1^, and their comparison exhibited the changes in the chemical composition of the uncoated versus coated samples. It can also be seen that the absorption band at 3621 cm^−1^ corresponds to H-O-H stretching vibration bands of water molecules weakly hydrogen bonded to the Si-O surface [32]. In the untreated sample, the peaks at 3278.2 cm^−1^ and 2894.9 cm^−1^ are attributed to stretching vibration of the OH group and asymmetric stretching of CH_2_ shifted in coated samples to 3263.8 cm^−1^ and 2890.6 cm^−1^ reported elsewhere [33]. The major reason stated for the stretching was believed to be the K band resonation of CH_2_ with approximate excitation energies of ~0.25 eV and de-excitation to the L band. In addition, a decrease in the intensity of the absorption peaks of coated samples compared to untreated samples can be observed. Notably, a small new peak at 1631 cm^−1^ appears in all coated samples, corresponding to the stretching vibration of carbonyl (C=O) [34]. Furthermore, the very strong absorption band at 1023.3 cm^−1^ signifies the Si-O bending vibration [35]. The Si-O bending vibration confirms the presence of the silicon-based compound in the coated samples, which is known for its thermal stability and provides a heat-resistant characteristic to the treated cotton. The small new peak at 614.8 cm^−1^ is attributed to the Al-O-Si bond [36].

Overall, the FTIR spectra confirm the significant chemical changes in the coated samples, which suggests that the coating has been conducted successfully.

### 2.3. TGA Analysis

**Figure 7:**
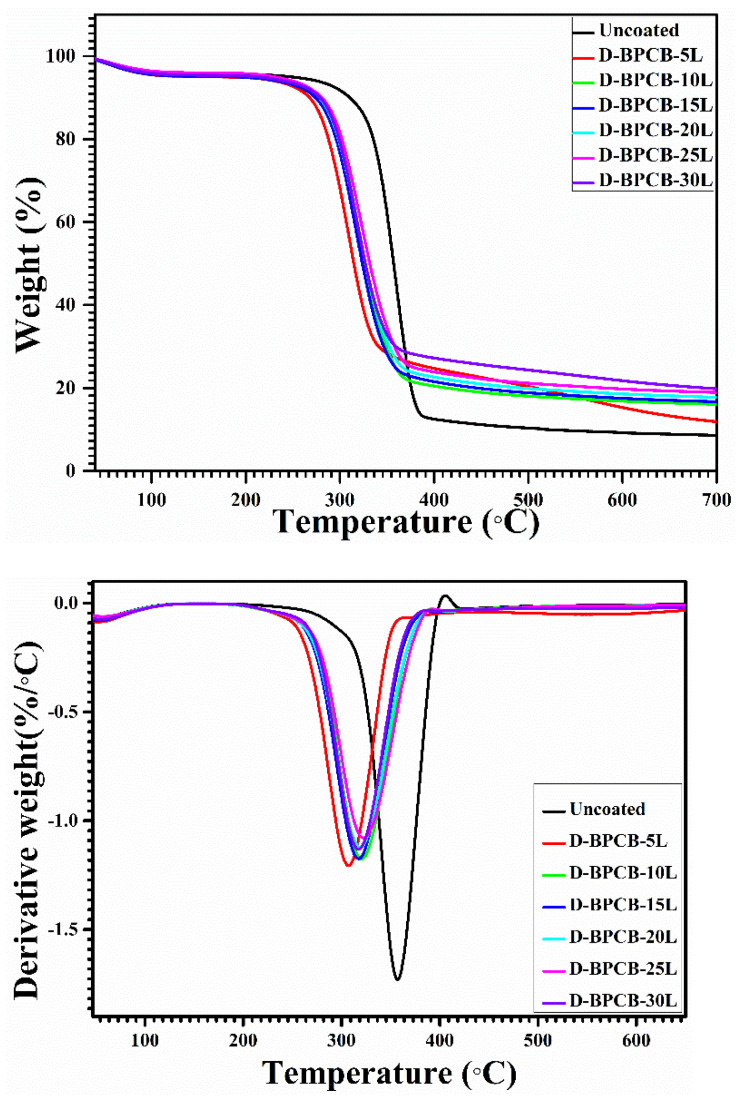
TGA and DTGA of coated and uncoated cotton samples.

The results from thermogravimetric analysis (TGA) and derivative thermogravimetric analysis (DTGA) plots are presented in Figure 7, with the corresponding data summarized in Table 1. Both the untreated and treated cotton fabric reported a single-step degradation process. At temperatures below 100 °C, minor weight loss was observed across all cotton samples, primarily attributed to the evaporation of moisture. For untreated cotton fibers, significant thermal degradation occurred within the temperature range of 310 °C to 390 °C. At this stage, maximum weight loss was observed at 360 °C, corresponding to a 59.9% reduction in mass. It is speculated that this higher degradation was largely caused by the dehydration and decarboxylation reactions of cellulose [37]. After 390 °C, the weight reduced gradually, leaving a residual mass of approximately 8.6% at 700 °C. However, in the layer by layer (LbL)-treated samples, the degradation process was found to be initiated at a much lower temperature (270 °C) than the untreated cotton samples, whereas the major degradation in 5BL–30BL samples was exhibited in the temperature range of 270 °C to 370 °C. As we discussed in earlier section, LbL-treated samples contain a significant portion of chitosan and BPEI. So, the degradation of treated samples at lower temperatures might be due to the degradation of chitosan and BPEI, which start degrading at temperatures of 247 °C and 300 °C, respectively [38,39,40]. Furthermore, the uncoated specimens left a final residual mass of nearly 8.6% at 700 °C, whereas in treated samples, the fire-retardant nature of chitosan and BPEI resists the mass degradation at higher temperatures (compared to the untreated cotton samples) and results in a higher residual mass. We observed a sequential increment in the residual mass with the number of immersions. The observed trend suggests that the amount of residue collected at the end of the thermal analysis is directly proportional to the deposited BL number, as listed in Table 1.

### 2.4. VFT Analysis

**Figure 8:**
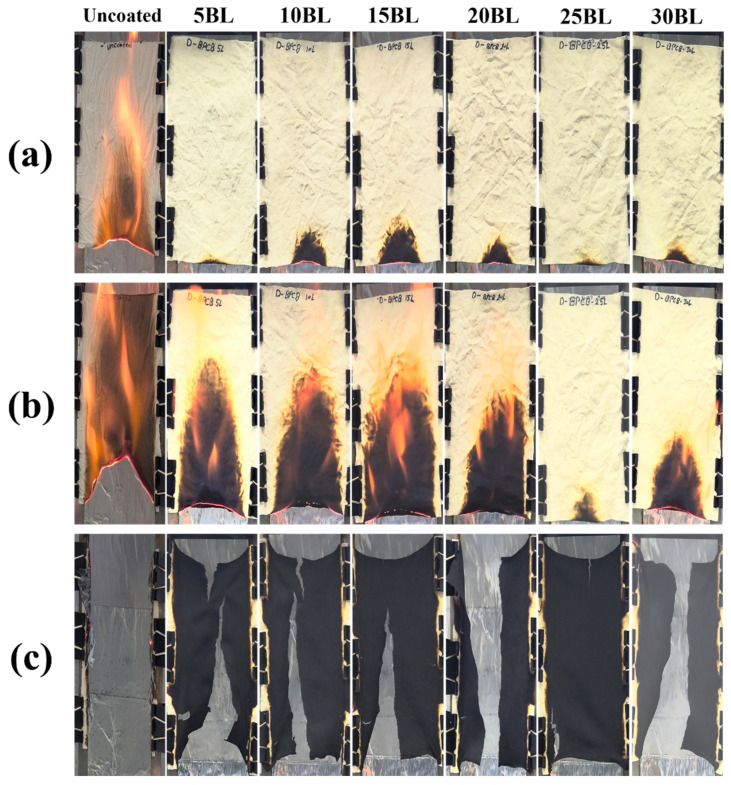
Images of the char residue of samples (**a**) after 5 s, (**b**) 10 s, and (**c**) final result of the vertical flame (UL-94) test.

The fire-retardant properties of the coated fabrics were precisely assessed by vertical flame test (VFT) measurements, conducted by exposing the samples to the tiny flame in accordance with the UL-94 standard. The objective is to link the macro-thermal characteristics with the micro-scale values acquired using TGA. However, the wash durability remains to be explored in future research. The VFT was performed on both untreated and coated samples, with optical images taken at 5 s and 10 s after the removal of the flame burner, as seen in the Figure 8. The final photos were captured after the complete combustion of all samples.

**Figure 9:**
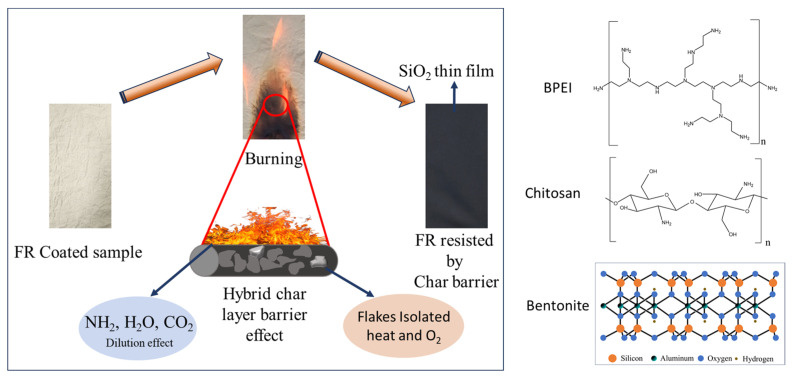
Schematic diagram of FR mechanism.

The results indicate that the untreated sample was highly flammable, as it ignited immediately upon direct contact with the combustion source. After it was ignited, the flame propagated rapidly through the untreated fabric, leading to complete combustion and almost no carbon residue remaining, transforming into gray-white ash. It can be observed that the no self-extinguishment was observed in the untreated sample. However, the flame propagation was effectively resisted by the coated samples. Schematic diagram of fire resistant is shown in Figure 9. From the size, intensity and effected area at specified time, it can be observed clearly that the 25BL resist the flame quite effectively at 10 s while the 5, 10, and 15BL weakly resist as compared to 20, 25 and 30BL samples.

The char obtained from the 25BL sample was most sustainable among all the samples, signifying its effectiveness against fire. Furthermore, their char structure remained intact, while other samples exhibited complete or partial structural breakage that may be due to strain induced by the combustion reactions. However, it can be seen that coating successfully suppressed the ignition initiation and flame spread. Also, it can be noted that self-extinguishment was achieved, but the flame spread behavior and ignition initiation were significantly retardant.

### 2.5. After Burn Surface Analysis

**Figure 10:**
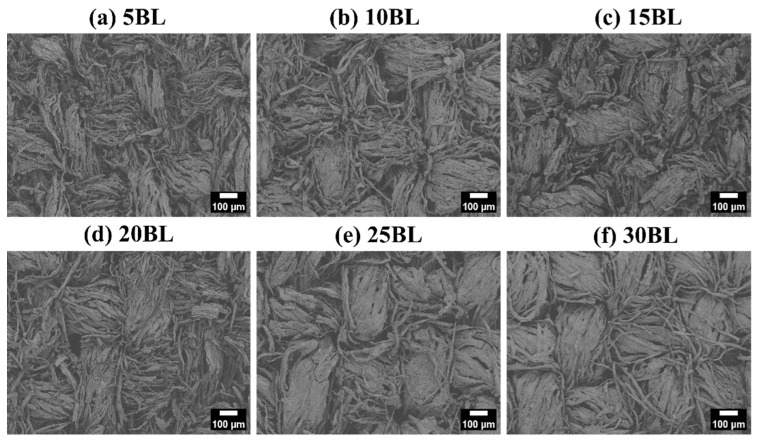
After burn LV-SEM surface morphologies of char residue.

To study the fire-retardant mechanism, the fabric residues after vertical flame test were examined by using LV-SEM. As shown in Figure 10, the cotton fibers exhibit significant fractured and shrinkage in diameter, leaving behind some slender and incomplete char residue after the test. Additionally, It can also be seen some loose granular remnants were attached to the surface, as reported in [41]. However, these granular remnants were mainly some inorganic SiO_2_ particles formed from the thermal degradation of Bentonite clay. The inorganic SiO_2_ particles work as a condensed phase barrier due to their high thermal stability, with a melting point of 1600–1700 °C. this barrier effectively protects the polymer matrix by forming a protective shield that enhanced the coated cotton’s resistance against fire at high temperature [42,43,44]. Moreover, during combustion, a compact and dense salicaceous char layer formed on the cotton fabric surface, effectively acting as a barrier to heat and oxygen transfer [45,46]. However, such an inorganic barrier may exhibit fire resistant behavior. Notably, as the number of deposited layers increases, the char residue retained a cohesive and solid structure. The residue is dense, non-porous and the original part of the material has been preserved. While some minor cracks were observed in the char structure, these are limited which indicating that small section of fiber have been affected.

**Figure 11:**
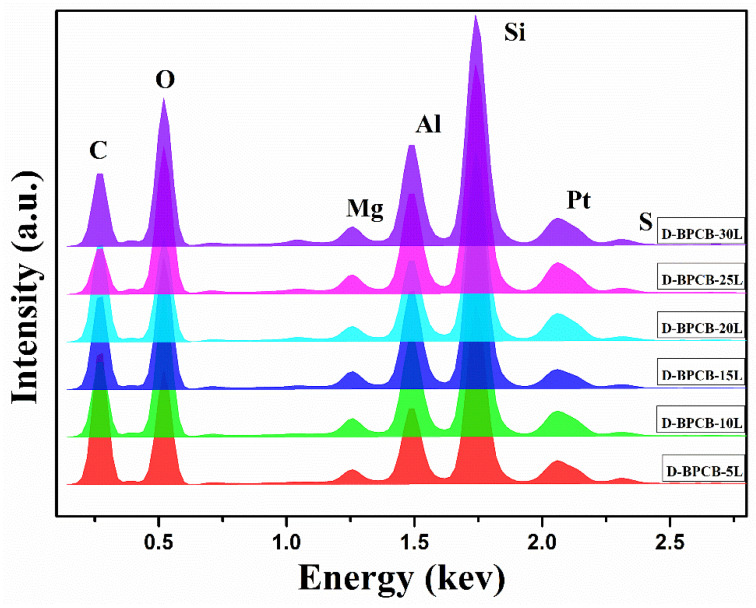
After burn EDS analysis of the char residue.

The EDX analysis of the char residue is shown in the Figure 11. The char residues of all of the coated samples showed a much higher Mg, Al, and Si content. The presence of greater quantities of these elements after burning suggest the effective role of these samples. Furthermore, the highest peak of “O” and “Si” confirms the active role of the SiO_2_ char layer.

## 3. Materials and Methods

### 3.1. Materials

The substrate cotton fabric (100%) was obtained from a local market in the Republic of Korea. Initially, the cotton fabric was cleaned by washing and then dried in an oven at 80 °C. The fabric was then cut into pieces at an approximate height and width of 210 mm and 110 mm, respectively. Bentonite clay (Al_2_O_3_·4(SiO_2_)·H_2_O), (BNT); chitosan (C_56_H_103_N_9_O_39_) with low molecular weight (5 × 10^4^–1.9 × 10^5^ Da), (CH); and branched polyethyleneimine H(NHCH_2_CH_2_)_n_NH_2_), (BPEI) were all purchased from Sigma Aldrich (St. Louis, MO, USA). Acidic acid, sulfuric acid 95% (H_2_SO_4_), sodium hydroxide 92% (NaOH), and DI water were also obtained.

### 3.2. Cationic and Anionic Solutions

All layer by layer (LbL) solutions were prepared using deionized (DI) water. The anionic bentonite clay (BNT) solution (2%) was prepared using DI water, and the pH was adjusted to 10 using a (2%) NaOH solution. For the cationic solution, chitosan (2%) was prepared in DI water and acetic acid and stirred overnight at 50 °C. Branched polyethyleneimine (BPEI) (2%) was prepared in DI water, heated to 80 °C, and finally reduced to ambient temperature for overnight stirring. A mixed solution of chitosan and BPEI was used for cationic deposition as a hybrid species. However, the pH of the hybrid solution was adjusted to 4.5 using a 1 M H_2_SO_4_ solution.

### 3.3. Layer by Layer Deposition Process

**Figure 12:**
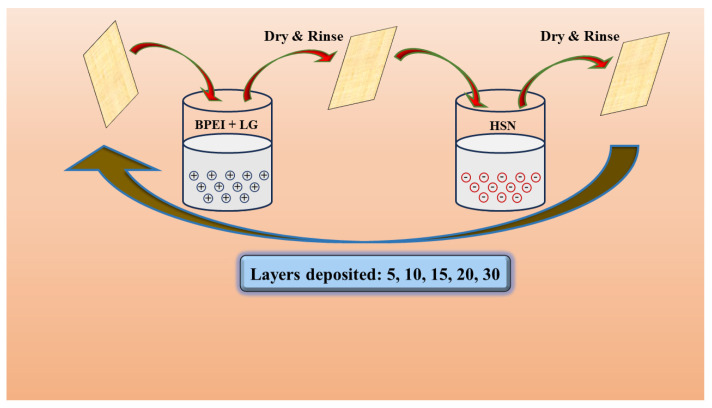
Schematic diagram of LbL-deposition.

The LbL deposition process consists of hand-dipping cotton fabric in an oppositely charged aqueous solution, as shown in Figure 12. The cotton fabrics were initially dipped in a cationic solution (BPEI, chitosan) for 5 min, dried at 100 °C, and then rinsed in DI water. Later, the samples were dipped in an anionic solution for 5 min, and then again dried and rinsed in DI water. Likewise, a single cycle was completed and named as one bilayer (BL). The cycle was repeated for 5, 10, 15, 20, 25, and 30 bilayers. After every 5BL, the solution pH was adjusted to 4.5 and 10, respectively. The initial BL time was 5 min, while the remaining BL took 1 min. The obtained dry branched polyethyleneimine chitosan bentonite clay (D-BPCB)-coated cotton fabrics were named as D-BPCB-5BL, D-BPCB-10BL, D-BPCB-15BL, D-BPCB-20L, D-BPCB-25L, and D-BPCB-30L.

### 3.4. Characterization

Surface morphologies were examined with a low-voltage scanning electronic microscope (LV-SEM, Merlin Compact, Carl Zeiss, Oberkochen, Germany). The char residue after VFT was also observed using LV-SEM. The elemental composition analysis of the coated samples and the post-VFT char residue was analyzed using EDX (Energy Dispersive X-ray). Before each analysis, the specimens were sputter-coated with platinum for around 2 min under a high vacuum environment. The micro-thermal characteristics of the specimens were assessed using a thermogravimeter analyzer (Perkin Elmer Pyris-1 instrument, Waltham, MA, USA) under nitrogen atmosphere (20 mL min^−1^). The temperature range for the analysis was 50–600 °C with a heating rate of ~20 °C min^−1^. The sample’s masses for each TGA experiment ranged from 10 to 15 mg. The macro-thermal characteristics of the pre- and post-coated specimens were assessed using vertical flame tests conducted in accordance with the ASTM D6413 standard. A custom-designed chamber was used to burn the samples, each measuring approximately 300 mm × 120 mm in size. A butane torch flame, maintained at a length of 20 mm, was directed towards the bottom of the fabric sample for 10 s to induce fire. The flame propagation, burn duration, and char formation were subsequently measured using a high-resolution optical camera (Samsung, Suwon, Republic of Korea).

## 4. Conclusions

In this work, a hybrid cationic solution and bentonite clay were deposited on cotton fabric (up to 30 bilayers). From the LV- SEM analysis, it was confirmed that the coating layers were successfully deposited on the substrate. With an increasing number of deposited layers, the fiber thickness also increased. FTIR analysis confirmed the chemical modifications in coated samples. The presence of SiO_2_ and Al-O-Si from the bentonite clay contribute to the formation of protective barriers against fire. VFT explored the efficiency of the coating, showing significant resistance to flame propagation. A higher content of Mg, Al, and Si in the char residue of coated samples shows the effective role of these elements during fire retardancy.

## Figures and Tables

**Table 1:** TGA and DTGA data of coated and uncoated samples.

Samples	T_−5%_ (°C)	T_−10%_ (°C)	T_peak_ (°C)	Residue (%) at T_700_ (°C)
Uncoated	247.5	310.4	360	8.6
D-BPCB 5L	169.8	267.6	306.9	11.9
D-BPCB 10L	209.7	277	320.3	16
D-BPCB 15L	185.1	273.8	317.2	16.7
D-BPCB 20L	228.8	277.9	318.6	17.8
D-BPCB 25L	230.8	280.7	322.3	18.9
D-BPCB 30L	213.5	278.6	316.8	19.8

## Data Availability

The research data are the sole property of the university.
